# Electrocardiographic features of children with Duchenne muscular dystrophy

**DOI:** 10.1186/s13023-022-02473-9

**Published:** 2022-08-20

**Authors:** Liting Tang, Shuran Shao, Chuan Wang

**Affiliations:** 1 Department of Pediatric Cardiology, West China Second University Hospital, Sichuan University, No. 20, 3rd section, South Renmin Road, Chengdu, 610041 Sichuan China; 2 The Cardiac Development and Early Intervention Unit, West China Institute of Women and Children’s Health, West China Second University Hospital, Sichuan University, Chengdu, Sichuan China; 3 West China Medical School of Sichuan University, Chengdu, Sichuan China; 4 Key Laboratory of Birth Defects and Related Diseases of Women and Children (Sichuan University), Ministry of Education Chengdu, Sichuan, China; 5 Key Laboratory of Development and Diseases of Women and Children of Sichuan Province, West China Second University Hospital, Sichuan University, Chengdu, Sichuan China

**Keywords:** Duchenne Muscular dystrophy, Electrocardiogram, Age, Genotype, Cardiac function

## Abstract

Duchenne muscular dystrophy (DMD) is a clinically common X-linked recessive myopathy, which is caused by mutation of the gene encoding dystrophin on chromosome Xp21. The onset of heart injury in children with DMD is inconspicuous, and the prognosis is poor once it develops to the stage of heart failure. Cardiovascular complications remain an important cause of death in this patient population. At present, population and animal studies have suggested that Electrocardiogram (ECG) changes may be the initial manifestation of cardiac involvement in children with DMD. Relevant clinical studies have also confirmed that significant abnormal ECG changes already exist in DMD patients before cardiomegaly and/or LVEF decrease. With increases in age and decreases in cardiac function, the proportion of ECG abnormalities in DMD patients increase significantly. Some characteristic ECG changes, such as ST-segment changes, T wave inversion, Q wave at the inferolateral leads, LBBB and SDANN, have a certain correlation with the indexes of cardiac remodeling or impaired cardiac function in DMD patients, while VT and LBBB have demonstrated relatively good predictive value for the occurrence of long-term DCM and/or adverse cardiovascular events or even death in DMD patients. The present review discusses the electrocardiographic features in children with DMD.

## Introduction

Duchenne muscular dystrophy (DMD) is a clinically common X-linked recessive myopathy, with an incidence in live male infants of approximately 1/3500–1/5000 [1–3]. DMD is characterized by symmetric myasthenia and amyotrophy, which gradually deteriorate, and is caused by mutation of the gene encoding dystrophin on chromosome Xp21. This gene is the largest known human gene, containing 79 exons [4–6]. Most children with DMD begin to exhibit abnormal gait at 3–4 years of age, gradually lose their walking ability at 10–12 years and die of circulatory and respiratory failure at 18–20 years of age [7–9]. With advances in modern medical technologies, the implementation of multidisciplinary management of DMD, early use of steroid hormones, application of nocturnal ventilation and early rehabilitation, and other treatments, the survival age of children with DMD can be extended to approximately 30 years [10, 11]. However, with this extended survival time, cardiovascular complications particularly the DMD-cardiomyopathy become increasingly prominent and account for the primary cause(s) of death among children with DMD.

Early detection of cardiac involvement using diagnostic technologies, quantitative evaluation of the degree of cardiomyopathy, and initiation of therapy in the early stage(s) of the disease may delay the progression of cardiac involvement and improve the overall prognosis of children with DMD [3]. Currently, clinical cardiac examination modalities mainly include electrocardiography (ECG), echocardiography, and cardiac magnetic resonance (CMR) imaging. Among these, ECG, which consists of general ECG, dynamic ECG and vectorcardiogram, affords the advantages of convenience, rapidity, low cost, and easy implementation. The ECG is a cornerstone in the diagnosis and management of cardiomyopathy. Traditionally, ECG abnormalities in cardiomyopathy were considered as non-specific. However, recent studies have demonstrated that ECG retains an extremely powerful role in the assessment of patients with both genetic and acquired forms of dilated cardiomyopathy (DCM).

Firstly, some ECG features are clues of specific DCM subtypes, as certain disease-causing genes are associated with characteristic ECG abnormalities that may have diagnostic value for the patients. For instance, the detection of paroxysmal supraventricular arrhythmias in young patients with DCM should prompt investigation for familial lamin A/C (LMNA) cardiomyopathy [12]. T-wave inversion, especially in the lateral leads, is a recognized feature of certain genetic forms (for example filamin C or desmosomal disease) [13]. Right bundle branch block (RBBB) and posterior/inferior Q waves are generally uncommon in patients with DCM but it is frequently found in patients with DMD [14]. Secondly, ECG changes may be the initial manifestation of cardiac involvement in patients with DCM. For example [15], in patients with variants of LMNA, early conduction disease, manifesting as sinus bradycardia, sinus node arrest, AV blocks (first or second degree AV block, later progressing to complete heart block) or left bundle branch block (LBBB), as well as atrial flutter, often precede the development of an overt dilated phenotype. T-wave changes and QTc prolongation may be useful as an early indicator before the onset of chemotherapy-related cardiac dysfunction in patients with doxorubicin-induced cardiotoxicity [15]. Similar findings were also observed in children with DMD [16, 17]. Most importantly, some specific ECG findings possess great predictive value in the cardiac dysfunction as well as the worse prognosis in cardiomyopathy. Prolonged QTc interval and decreased QRS voltages have been shown to correlate with LV systolic dysfunction in patients with DCM caused by drugs and toxins [18]. The presence of complete LBBB (CLBBB) [19, 20], fragmentation of the QRS complex [21], low QRS amplitude [22, 23] and anterolateral T wave inversion [24] as well as non-sustained ventricular tachycardia [12] were widely identified as major risk factors for sudden cardiac death and all-cause mortality in patients with DCM. In addition, heart rate variability (HRV) obtained from dynamic ECG (DCG) analysis has been used in multiple diseases as a measure of cardiac autonomic activity and has been associated with various morbidity and mortality outcomes [25, 26]. In terms of the vectorcardiogram, both the spatial and frontal QRS-T angle not only have prognostic values on development of cardiovascular events but also have implications on cardiac functional performance. The frontal QRS-T angle has additionally been associated with ventricular remodeling and function in patients with heart failure with preserved ejection fraction [27–35].

In summary, a systematic analysis of the ECG could provide diagnostic red flags useful to orient the following phases of the diagnostic work-up, prognostic stratification criteria and information that can direct appropriate decision making in patients with cardiomyopathy. In the past decades, increasing numbers of studies have explored the characteristics of ECG in patients with DMD. Accumulating evidences have demonstrated that ECG can be used to diagnose arrhythmia and preliminarily assess the scope of myocardial damage in children with suspected DMD, thereby providing important clues for clinicians to confirm the disease and judge prognosis. More importantly, population and animal studies have suggested that ECG changes may be the initial manifestation of cardiac involvement in children with DMD. Before myocardial fibrosis appears in the heart, ECG can reveal abnormal signs. ECG may be more sensitive than echocardiography—and even CMR imaging—in detecting early manifestations of DMD-related myocardial involvement [17, 36]. Therefore, this review was carried out (1) to discuss the underlying mechanism(s) of abnormal cardiac electrical activity caused by DMD; (2) to summarize the characteristics of ECG findings in patients with DMD; (3) to analyze the factors influencing ECG abnormalities in patients with DMD; (4) to find clues regarding the relationship between ECG abnormalities and cardiac function as well as the prognosis in patients with DMD.

## Methods

### Eligibility

Cross-sectional, prospective, or retrospective studies with ≥ 10 patients, published as full articles in PubMed and CNKI were included. Studies published in the form of abstracts, or with < 10 subjects were excluded.

### Search strategy

**Fig. 1:**
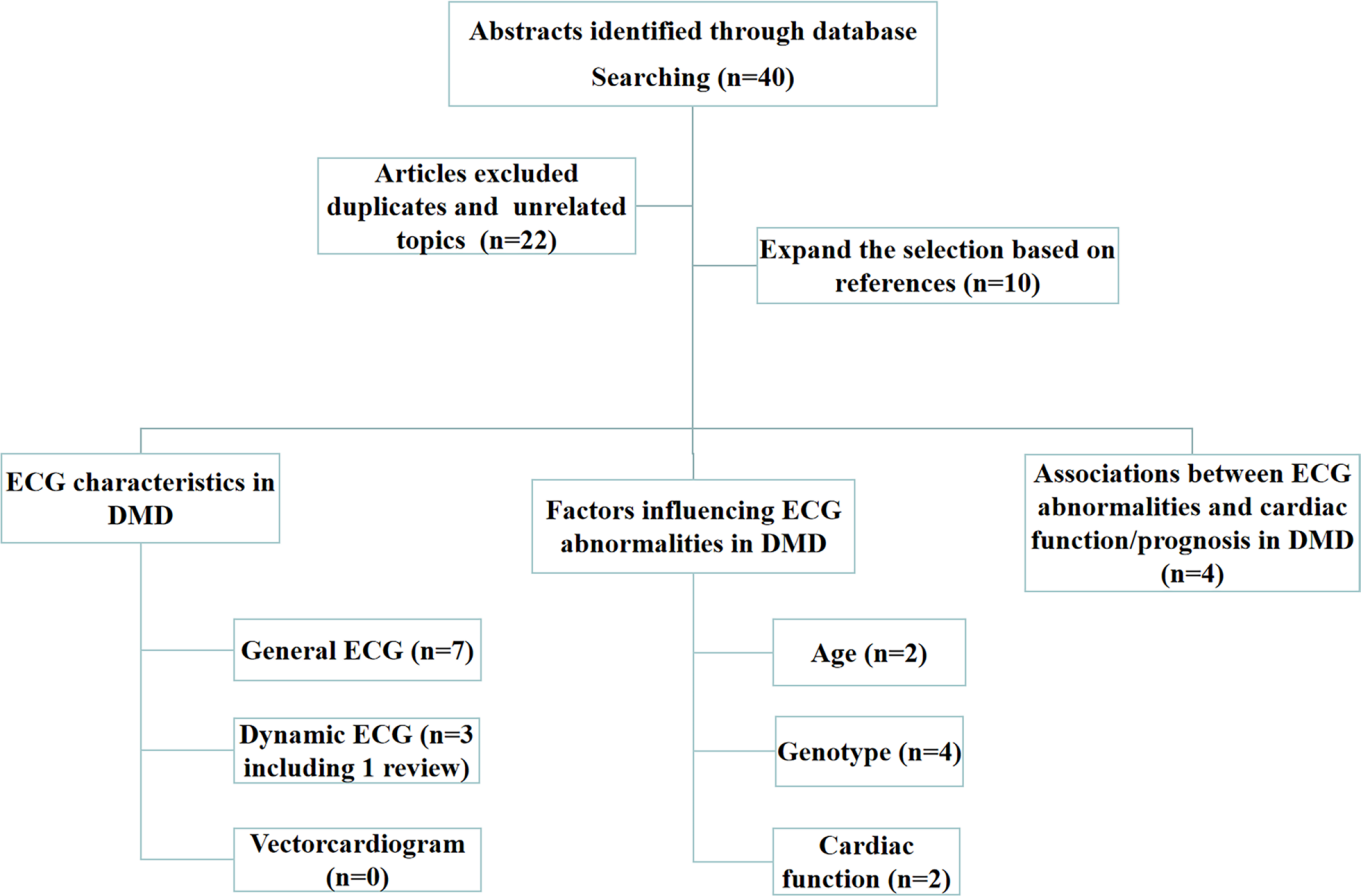
Flow diagram illustrating the study selection process for search criteria

These articles were divided into three groups for retrieval: (1) study the electrocardiogram changes in DMD patients, including General electrocardiogram, dynamic electrocardiogram and vectorcardiogram; (2) study the factors influencing ECG abnormalities in DMD,such as age,genotype and cardiac function; (3) investigate the associations between ECG abnormalities and cardiac function/prognosis in DMD. Then full-text analysis of findings within eligible articles. The scope of retrieval was expanded through targeted references, and 28 articles were included after Eligibility examination. All abstracts were reviewed by Liting Tang and checked by Chuan Wang. Disagreement was resolved between the two reviewers. Full texts of eligible articles were retrieved for review. The research selection process of search criteria is shown in Fig. 1.

## Underlying mechanism(s) of abnormal cardiac electrical activity caused by DMD

### Cardiomyocyte inflammation, necrosis, and fibrosis

Dystrophin is a cytoskeletal protein distributed on the plasma membrane of skeletal muscle cells and cardiomyocytes. It plays the role of cell scaffold, maintains the integrity of muscle fibers, and protects against contraction-induced damage [37]. Loss or lack of dystrophin leads to the rupture of muscle membranes during contraction, leading to increases intracellular calcium ion levels, which in turn triggers a cascade reaction in cells, leading to inflammation, necrosis, and fibrosis. On one hand, the increase in intracellular calcium concentration is related to an increase in leakage in calcium channel activity on the cell membrane surface and, on the other hand, is caused by the increase in inactivated calcium channels in malnourished cardiomyocytes [38]. The increase in intracellular calcium concentration in cardiomyocytes activates proteolytic enzymes and phospholipases, leading to degradation of cellular phospholipids, and produces hemolytic glycerophospholipids, which exert significant electrophysiological toxicity [39]. In 1977, Sanyal et al. [40]. explored myocardial ultrastructure in DMD patients in the United States. They found that, compared with normal cardiomyocytes, the number of mitochondria in cardiomyocytes in malnourished areas increased exhibited a series of structural changes, including slight swelling, partial loss or interruption of cristae, and occasional appearance of electron-dense bodies. Whether in skeletal muscles or myocardium, myofibrils were lost, which induced muscle fibers to exhibit a “worm-eaten” appearance. Subcellular changes in patients with DMD often involve the sub-epicardium of the posterior basal part of the left ventricle, followed by the posterior papillary muscle, ventricular septum, and right ventricular free wall. At the same time, changes in the right atrium or left atrium are very minor, which could explain the fact that the most common abnormal ECG changes in children with DMD are deep Q waves at the anterolateral wall leads [40].

In 1982, Sanyal et al. [41] systematically described the morphological characteristics of the conduction system in 3 patients with DMD in Saudi Arabia to identify factors affecting cardiac electrical activity. Compared with an age- and sex-matched normal control group, the hearts of these 3 patients exhibited multifocal degenerative changes, characterized by vacuolation, fatty infiltration, karyopyknosis, muscle fiber loss, and moderate to severe fibrosis. These malnutrition-related changes are similar in all patients and involve the atrial preferential pathways of the sinus node and atrioventricular node, the upper segment of the atrioventricular node, His bundle, sub-endocardium, and infranodal left and right bundle branches.

### Loss of dystrophin in the membrane of Purkinje fiber cells

Animal experiments have confirmed that abnormal ECG changes in animals with muscular dystrophy occur earlier than myocardial fibrosis [36] (i.e., ECG changes appeared without cardiomyocyte necrosis or fibrosis), suggesting that there may be other mechanisms underlying ECG changes in children with DMD. Research has shown that this may be related to the deletion of dystrophin in the cell membrane of Purkinje fibers, which are a subset of the various cell types comprising the cardiac conduction system [42].

In 1992, American investigators Bies et al. [39] first confirmed the expression and localization of dystrophin in the Purkinje fibers of the human heart and highlighted the importance of dystrophin in the function of Purkinje fibers of the heart. Subsequently, Bies et al. [39] also found that dystrophin is located on the membrane surface of Purkinje fibers in normal individuals, and its absence would lead to dysfunction of the cardiac conduction system. Kazuhiko Sega speculated that the absence of dystrophin in the conduction system would lead to left axis deviation and a shortened PR interval [43]. Ishikawa believed that Purkinje fiber blockage was the pathophysiological basis of the QRS notch [44]. More importantly, the canine X-linked muscular dystrophy (CXMD) model established by Jeanne James [45], found that in 4-month-old dogs with muscular dystrophy, when myocardial fibrosis had not yet appeared in the posterior basal segment of the left ventricle and cardiac function was still normal, the Purkinje fibers already exhibited significant vacuolar degeneration. The results of electron microscopy revealed that virtually all of the organelles and myofibrils in Purkinje fibers disappeared, and broken mitochondria and myofibrils were near the vacuoles [17]. Therefore, we speculate that deletion of dystrophin in the membrane of Purkinje fibrocytes is the pathophysiological basis of early-stage ECG abnormalities in children with DMD. As such, ECG may be more sensitive than echocardiography and even CMR imaging in detecting early manifestations of DMD-related myocardial involvement.

## Abnormal ECG findings in children with DMD

### General ECG

Several studies have investigated the characteristics of routine 12-lead ECG in children with DMD. In 2008, Takami et al. [46] retrospectively analyzed 136 ECG reports from 69 children (4–18 years of age) in Japan with genetically confirmed DMD and found that 91.3% (64/69) of the reports documented ≥ 1 abnormal ECG manifestation(s). Among these, the most common was deep Q waves (55.1%), which were mostly at the anterolateral wall leads (I, avL, and V4-V6) (53.6%), and could also appear at the inferior wall leads (II, III, and avF) (15.9%). Other common abnormal ECG manifestations included R/S ratio at lead V1 (37.7%), high R wave amplitude at lead V1 (36.2%), short PR interval (33.3%), QRS notch (33.3%), low amplitude of RV5 + SV1 (24.6%), and sinus tachycardia (17.9%). Only one child exhibited prolonged QT interval, and no conduction block was reported. In 2009, a study from the United States [47] retrospectively analyzed 115 ECG reports from 115 children with DMD (3.6–27.8 years of age), among which the most common manifestation was short PR interval (43%), followed by right ventricular hypertrophy (37%), low or inverted T waves (33.0%), and deep Q waves (the incidences at different leads varied from 10 to 34%, and were highest at V5-V6), while no QT interval prolongation or conduction block was reported. In 2010, Shah et al. [16] retrospectively analyzed 377 ECG reports from 150 children with cardiomyopathy (including 86 children with DMD, with an average age of 10.1–16.9 years) in the United States, and found that 71% (269/377) exhibited ECG abnormalities, the most common of which was nonspecific T wave changes (28%), while others included right ventricular hypertrophy (21%), left ventricular hypertrophy (11%), Q waves at the inferolateral leads (10%), intraventricular block (8%), left axis deviation (8%), biventricular hypertrophy (7%), T wave inversion at the inferolateral leads (7%), and ST-segment depression (5%), with 4 ECG reports documenting prolonged QT interval (1%), and 8 documenting complete right bundle branch block (CRBBB) (2%). In 2011, James et al. [17] analyzed 78 ECG reports from 78 children with DMD < 6 years of age (range, 0.2–5.7 years) who were not treated with hormone(s), and found that 78% exhibited at least one abnormal ECG manifestation(s), among which left ventricular changes were the most significant (52/77 [68%]), with Q wave (III, V6) being the most common (57%), followed by increased R/S ratio at lead V6 (19%) and elevated R wave at lead V6 (14%). Right ventricular changes accounted for 22% (17/77), among which elevated R wave at lead V1 was the most common (16%). Biventricular changes were observed in 17% of the children. Pathological changes in the ST-segment and T wave were found in 3% of the children. Sinus tachycardia, prolonged QT interval, and short PR interval each accounted for 4%. No conduction block was reported. In 2017, another study from Japan [43] included 47 DMD patients (27.6 ± 6.0 years of age) and analyzed 400 ECG reports before enrollment. The results revealed that PR interval and QRS interval widened with age, 5 patients experienced CRBBB, 2 complete left branch bundle block (CLBBB), 5 nonspecific intraventricular blocks, and 1 complete atrioventricular block (cAVB) at 28 years of age; however, cardiac function and heart size were normal. In 2018, Fayssoil et al. [48] enrolled 121 adult DMD patients (18–41 years of age) in France, among whom 95% required respiratory support. The authors found that 34% (41/121) of the patients experienced RBBB and 13% (15/121) experienced LBBB. Supraventricular tachycardia (SVT) was found in 6% of patients, and ventricular tachycardia (VT) in 2%. In 2018, investigators from Fudan University in China [49] retrospectively analyzed 270 ECG reports from 246 children (2 months to 15 years of age) with DMD in the Chinese population and found that the rate of ECG abnormalities was 22.96% (62/270). Common abnormal changes included left ventricular hypertrophy/high voltage, Q wave (mainly distributed at leads II, III, aVF, and V4-V6), deepened S wave at lead V1, R/S ratio at lead V1 higher than the normal range, right ventricular hypertrophy, and ST‐T segment changes, among which left ventricular hypertrophy/high voltage, deepened S wave at lead V1 and abnormal Q wave, which reflected manifestations of left ventricular electrophysiological changes, accounted for 66.13% (41/62) of abnormalities in ECG reports. These details were summarized in Table 1.

**Table 1:** Summary of studies that investigate characteristics of general ECG in patients with DMD

Year	Inclusion criteria	Exclusion criteria	Study type	Sample	Total ECG	Age	LV ECG changes	Abnormal Q wave	Low amplitude of RV5 + SV1	ST-T segment changes	Short PR interval	Prolonged QT interval	Conduction block	Sinus tachycardia	Others
2008	DMD < 18	Steroids, ACEI used before ECG	Retrospective	69	136	4–9	None	54.50%	3.00%	None	33.3%	Exist	None	17.9%	Total abnornal ECG 91.3% (63/69)
						10–13		46.20%	23.10%						
						14–18		85.70%	50.00%						
2009	EF < 55%	–	Retrospective	115	115	3.6–27.8	None	10–34%	None	33.0%	43.0%	None	None	None	–
2010	DMD (2004–2009)	–	Retrospective	150	377	10.1–16.9	None	10%	None	33.0%	None	1.0%	10%	None	–
2011	DMD < 6	Not treated with hormones	Retrospective	78	78	0.2–5.7	None	57.0%	None	3.0%	4.0%	4.0%	None	4.0%	19% (increased R/S ratio at V6)
2017	DMD > 20	–	Retrospective	47	386	27.7 ± 6.0	None	None	None	None	None	None	5 CRBBB; 2 CLBBB, 5 indoor block; 1 cAVB	None	–
2018	DMD > 18	–	Retrospective	121	121	18–41	None	None	None	None	None	None	34% with RBBB; 13% with LBBB	None	6% with SVT; 2% with VT
2018	DMD (2011–2016)	Cardio dysfunction	Retrospective	246	270	0–3	7.89%	1.61%	None	None	None	None	None	None	11.8%(9/76)
						4–6	10.53%	1.61%							18.4%(14/76)
						7–9	19.77%	8.10%							26.7%(23/86)
						≥ 10	7.89%	1.61%							50.0%(16/32)

*Abbreviations*: DMD: Duchenne muscular dystrophy; ECG: electrocardiogram; EF: ejection fraction; LBBB: left bundle branch block; CLBBB: complete left branch bundle block; CRBBB: complete right bundle branch block; cAVB: complete atrioventricular block; ACEI: ansgiotensin-converting enzyme inhibitor

In summary, we found that the incidence of 12-lead ECG abnormalities in children with DMD were quite different across studies, and several potential explanations are supposed. First, the age ranges of the study populations were different, with several studies confirming that the incidence of ECG abnormalities in children with DMD increases with age. Second, there were differences in geographical regions and race among the study populations. Third, different diagnostic criteria for ECG abnormalities: some studies included all non-specific T-wave changes in ECG abnormalities, which may lead to the overestimation of ECG abnormalities in children with DMD. Although the types and incidences of ECG abnormalities in children with DMD were quite different across studies, there were some consistent findings. Pathological Q waves at leads I, II, III, avL, avF, and V4-V6, increased R wave amplitude with increased R/S ratio at lead V1, right ventricular hypertrophy, left ventricular high voltage, and ST-segment and T wave changes were common abnormal ECG changes in children with DMD. Sinus tachycardia and short PR interval were common and characteristic ECG abnormalities in children with DMD. Conduction block, including CRBBB, CLBBB, and cAVB, was not common in children with DMD, and most occurred in older children with DMD. To date, only 4 cases of cAVB in children with DMD have been reported in the literature, and these were detected at 28, 30, 33, and 40 years of age, respectively [50–52] QT interval prolongation was rare. SVT and VT may occur; however, most occurred in older DMD patients with impaired cardiac function.

### Holter dynamic electrocardiography

DCG can continuously record the entire process of cardiac electrical activity for 24 h and can identify arrhythmia(s) and myocardial ischemia, which are not easy to detect using conventional surface ECG examinations. Presently, a few studies have investigated DCG in patients with DMD. In 2010, Shah et al. [16] retrospectively included 150 children with myopathy (86 children with DMD, average age, 10.1–16.9 years) in the United States. Among these, there were 64 DCG reports (45 children), of which 25 children had dilated cardiomyopathy (DCM), and 20 had no DCM. The overall incidence of tachycardia was 27% (17/64). The incidence of tachycardia in the DCM group was significantly higher than that in the non-DCM group. Only one child without DCM had tachycardia (non-paroxysmal atrial tachycardia). Among the 17 cases of tachycardia, 2 children had atrial tachycardia, 1 had atrial flutter, 2 had re-entrant tachycardia, and 12 had VT. The 12 DMD children with VT were all diagnosed with DCM before VT, suggesting that VT is more likely to occur in children who have progressed to DCM and also have left ventricular systolic dysfunction. There were also 5 children with frequent premature ventricular contraction, all of whom had progressed to DCM before they were found to have premature ventricular contraction. VT is a risk factor for death in children with DMD. The 6 children with VT died 0.68 ± 0.41 years after VT occurred. Among them, 4 died of end-stage heart failure, 1 of unknown cause, and 1 of pneumonia and sepsis complications. An implantable cardioverter-defibrillator (ICD) was placed in 3 children with VT; nevertheless, they still experienced paroxysmal VT after a follow-up of 6.4 ± 3.7 months, although all survived or did not undergo additional treatment. In 2016, American researcher Villa [53] conducted a retrospective analysis of DMD patients who underwent DCG between 2010 and1 2014. The study included 442 DCG reports of 235 patients (mean age, 14 ± 4 years), and divided them into three groups according to left ventricular ejection fraction (LVEF): ≥ 55% (n = 184); 35%–54% (n = 46); and < 35% (n = 5). The results revealed that ectopic atrial rhythm was relatively common (68%), frequent premature atrial contraction and non-sustained atrial tachycardia were more common in patients with cardiac insufficiency, and none of the patients experienced sustained atrial tachycardia. In addition, ectopic ventricular rhythm was also common (45%), and ventricular arrhythmia was also more common in patients with cardiac insufficiency. Among patients in the LVEF < 35% group, 30% had non-sustained VT, while the incidences of VT in the EF ≥ 55% group and EF 35%–54% group were 0% and 2%, respectively. In addition, compared with asymptomatic patients, those with symptoms demonstrated a higher incidence of DCG abnormalities. These studies were illustrated in Table 2.

**Table 2:** Summary of studies that Dynamic ECG for prediction of abnormal cardiac function

Year	Inclusion criteria	Exclusion criteria	Study type	Sample size	Total ECG	Age enrollment	EF abnormality	ECG abnormality	VT	others
2010	DMD(2004–2009)	None	Retrospective	150	377	10.1–16.9	Not specified	94% DCM with ST changes	27% with VT in DCM, 6 died after 0.68 ± 0.41 years later	5 with abnormal ECG progressed to DCM in 3.7 ± 2.6 years
2016	DMD(2010–2014)	None	Retrospective	235	442	11.0–17.0	LVEF ≥ 55%	Not specified	0	Those with symptoms demonstrated a higher incidence of DCG abnormalities
							LVEF = 35%-54%		2%	
							LVEF < 35%		30%	

*Abbreviations*: DMD: Duchenne muscular dystrophy; ECG: electrocardiogram; EF: ejection fraction; LVEF: left ventricular ejection fraction; VT: ventricular tachycardia; LBBB: left bundle branch block; DCM:Dilated cardiomyopathy

DCG can also be used to analyze heart rate variability (HRV), which can reflect the activity of the autonomic nervous system and quantitatively evaluate the tension and balance of cardiac sympathetic nerves and vagus nerves, and be used as a valuable predictor of adverse cardiovascular events. Presently, commonly used methods for HRV analysis include time-domain and frequency-domain analyses [54]. Time-domain analysis is used to measure HRV in a time domain using statistical methods. The principle of the frequency domain analysis method is to decompose randomly changing RR interval or instantaneous heart rate signal into frequency domain components with different energies for analysis, which can simultaneously evaluate the level of sympathetic and vagus nerve activity. Time-domain indexes include the standard deviation of normal-to-normal RR intervals over 24 h (SDNN, ms), standard deviation of all 5 min RR interval means (SDANN, ms), mean of the standard deviation of each 5 min RR interval mean (SDNN Index, ms), root mean square of successive differences between adjacent RR intervals in the whole course (RMSSD, ms), and percentage of beats with difference > 50 ms between adjacent RR intervals in total beats (pNN50, %). Frequency domain indexes include high-frequency power (HF, 0.15–0.4 Hz) which reflects vagal tone activity, low-frequency power (LF, 0.04–0.15 Hz) which reflects sympathetic tone activity, and LF/HF ratio [25]. In 2018, da Silva et al. [53] conducted a meta-analysis of current research on HRV in patients with DMD, which included 8 studies with sample sizes ranging from 17 to 124. Analysis revealed that, compared with the control group, SDNN, RMSSD and pNN50 decreased, HF decreased, and LF and LF/HF increased in DMD patients, and these changes appeared before LVEF decreased, suggesting that in DMD patients, HRV decreased, cardiac autonomic regulation was impaired, vagus nerve tension activity decreased, and sympathetic nerve activity increased.

The etiology of HRV alterations in DMD patients remains speculative and several explanations were hypothesized. Firstly, previous studies have shown alteration of nitric oxide and vascular endothelial growth factor secretion in the myocardium of dystrophin deficit mice. Nitric oxide has been shown to play important vagal tone by direct and agonistic effect in preganglionic and postganglionic neurons. Therefore, nitric oxide alteration might play a role in the changes of HRV in patients with DMD [25, 55–57]. Secondly, using Positron Emission Tomography, hypometabolism has been documented in temporal gyri, uncus, cerebellum and hippocampus in patients with DMD [58]. Interestingly, cardiac gated fMRI study which correlated HRV measures with brain area has shown that above areas have prominent influence of autonomic modulation [59]. In addition, histopathological studies of cortex of DMD patients have revealed impairments, particularly loss of neurons, gliosis, dentritic aberration, astrocytosis and perinuclear vacuolation [26]. Thus, central autonomic dysregulation might influence the alteration in HRV values in DMD. Thirdly, the study by Thomas et al. [60, 61]. demonstrated that the SDANN was significantly associated with positive Late Gadolinium Enhancement (LGE) on cardiac Magnetic Resonance, suggesting the persistent activation of the sympathetic nervous system seems to be a driving force in the pathological formation of myocardial fibrosis. Lastly, Mochizuki et al. proposed that abnormally low CVrr (coefficient of variation of RR interval < 3%) results from respiratory insufficiency-induced hypercapnia in patients with DMD [62]. Lanza et al. [63]. found a weak but significant correlation between autonomic impairment and degree of respiratory failure. Potentially corroborating these findings, Reis et al. [64]. showed that cardiac autonomic control of heart rate was associated with inspiratory muscle weakness in coronary heart failure. Nevertheless, any causality between respiratory and autonomic dysfunction remains only putative, and the two may instead derive from a common unidentified mediator. Taken together, these data suggest that impaired cardioautonomic regulation in patients with DMD likely results from multiple components, potentially including not only respiratory and mechanical cardiac dysfunction, but other factors such as structural and functional abnormalities of the sinoatrial node, neurohumoral, changes caused by inactivity of patients, and abnormal mechanoreceptor- and metaboreceptor-mediated reflexes originating from the diseased skeletal muscles.

In summary, DCG can detect ECG changes in children with DMD more comprehensively, including various types of atrial arrhythmia and ventricular arrhythmia, although the overall incidence rate varies significantly across studies. Cardiomegaly or impaired cardiac function, especially LVEF < 35%, is a predictor of abnormal performance in DCG. In addition, children with clinical symptoms are more likely to exhibit abnormal changes in DCG. Therefore, for those with clinical symptoms and/or cardiomegaly and/or decreased LVEF, DCG monitoring should be strengthened to identify abnormalities early and improve the prognosis of children with DMD. Moreover, children with DMD experience cardiac autonomic dysfunction and increased sympathetic nerve activity; as such, whether HRV-related indexes can predict the long-term adverse prognosis of children with DMD remains to be further explored.

## Factors influencing ECG abnormalities in children with DMD

### Age

Almost all studies have confirmed that older age is an important risk factor for increased ECG abnormalities in children with DMD. In 2008, Takami et al. [46] retrospectively analyzed 136 ECGs from 69 children in Japan with DMD (4–18 years of age). After stratified by age subgroups (4–9, 10–13, and 14–18 years), it was found that the incidence of ECG abnormalities increased significantly with older age: 84.8% of children < 10 years of age exhibited at least one type of ECG abnormality, while all children > 13 years of age suffered from different types of ECG abnormalities. In addition, after subgroup analysis of the types of ECG abnormalities, it was found that deep Q wave (54.5% versus [vs] 46.2% vs 85.7%) and low amplitude of RV5 + SV1 (3.0% vs 23.1% vs 50.0%) increased significantly with age, while other types of ECG abnormalities exhibited no significant difference across the age groups, suggesting that deep Q wave and low RV5 + SV1 amplitude may be the early signs of cardiomyopathy in children with DMD. However, whether deep Q wave and low RV5 + SV1 amplitude can predict the occurrence of cardiomyopathy in children with DMD remains to be further explored. In 2018, investigators from Fudan University in China [49] retrospectively analyzed the characteristics of 270 ECG from 246 children with DMD (2 months to 15 years of age) and reported an ECG abnormality rate of 22.96% (62/270). The rate of ECG abnormalities in all age groups increased gradually with age and were 11.84% (9/76), 18.42% (14/76), 26.74% (23/86), and 50.00% (16/32) in the 0–3, 4–6, 7–9 and ≥ 10 years age groups, respectively. There were statistically significant differences in ECG abnormalities of left ventricular hypertrophy/left ventricular high voltage and ST‐T changes among the different age groups (*P* = 0.024). ECG manifestations reflecting left ventricular electrophysiological changes, including left ventricular hypertrophy/left ventricular high voltage, deepened S wave at lead V1, and abnormal Q wave, accounted for 66.13% (41/62) of total ECG abnormalities, among which the proportion in each age group, in sequence, was 7.89%, 10.53%, 19.77%, and 31.25%, demonstrating an increasing trend with age. In addition, conduction blocks, such as CRBBB, CLBBB, cAVB, SVT, and VT, were also more common in older children, especially in children with DMD and impaired cardiac function.These findings are summarized in Table 3.

**Table 3:** Summary of studies that age related ECG abnormalities

Year	Inclusion criteria	Exclusion criteria	Study type	Sample	Total ECG	Age	LV ECG changes	Abnormal Q wave	Low amplitude of RV5 + SV1	ST-T segment changes	Short PR interval	Prolonged QT interval	Conduction block	Sinus tachycardia	Others
2008	DMD < 18	Steroids, ACEI used before ECG	Retrospective	69	136	4–9	None	54.50%	3.00%	None	33.3%	Exist	None	17.9%	Total abnornal ECG 91.3% (63/69)
						10–13		46.20%	23.10%						
						14–18		85.70%	50.00%						
2018	DMD (2011–2016)	Cardio dysfunction	Retrospective	246	270	0–3	7.89%	1.61%	None	None	None	None	None	None	11.8%(9/76)
						4–6	10.53%	1.61%							18.4%(14/76)
						7–9	19.77%	8.10%							26.7%(23/86)
						≥ 10	7.89%	1.61%							50.0%(16/32)

*Abbreviations*: DMD: Duchenne muscular dystrophy; ECG: electrocardiogram; EF: ejection fraction

### Genotype

The onset of heart injury in children with DMD is inconspicuous and the prognosis is not favorable once it progresses to heart failure. Understanding relationship between the ECG characteristics and genotype in children with DMD may be useful in detecting early cardiomyopathy in children with DMD and predicting prognosis in clinical practice. Several studies have investigated the correlation between genotype and ECG abnormalities in children with DMD. In 2008, Takami et al. [46] retrospectively analyzed 136 ECG graphs from 69 children with DMD (4–18 years of age) in Japan and found no significant correlation between ECG abnormalities and genotype. Subsequently, in the United States, James et al. [17] analyzed 78 ECG reports from 78 children < 6 years of age (range, 0.2–5.7 years) who did not undergo hormone treatment and found no significant difference between ECG abnormalities and genotype. In addition, in 2018, Fayssoil et al. [48] studied 121 DMD patients (18–41 years of age) in France. They found that 34% (41/121) of patients had RBBB and 13% (15/121) had LBBB; however, the occurrence of LBBB was not related to genotype. Different from the above research, investigators from Fudan University in China [49] retrospectively analyzed the characteristics of 270 ECG graphs from 246 children with DMD (2 months to 15 years of age). The distribution of gene exon deletion in children with DMD was mainly concentrated in exons 3–21 and 45–52, and the rates of ECG abnormalities in the exons 1–2 and 44–45 deletion groups demonstrated statistically significant differences from groups with deletion(s) at other loci [49, 65]. In summary, in view of the many positions and types of dystrophin gene mutations in children with DMD, the relevant research samples are all small, and the correlation between dystrophin gene mutations and ECG abnormalities has not been determined and, thus, warrants further exploration.

### Cardiac function

Impaired cardiac function is a risk factor for various types of arrhythmia among cardiomyopathies caused by various factors and DCM caused by DMD is no exception. In 2010, Shah et al. [16] retrospectively analyzed 377 common ECG reports from 150 children with myopathy (including 86 with DMD, with an average age of 10.1–16.9 years) in the United States. The children were divided into the DCM group (n = 64) and non-DCM group (n = 86) according to the presence of cardiomyopathy (LVEDD >  + 2 Z score and/or EF < 55%). It was found that the incidence of ECG abnormalities in the DCM group (94% [60/64]) was significantly higher than that in the non-DCM group (44% [38/86]) (*P* < 0.001). In their study, 45 children underwent 64 DCG examinations, 25 had DCM, and 20 had no DCM. The incidence of tachycardia in the DCM group was significantly higher than that in the non-DCM group, and only 1 child without DCM had tachycardia (non-paroxysmal atrial tachycardia). In addition, a study from the United States [53] included 442 DCG reports from 235 DMD patients (mean age, 14 ± 4 years). Patients were divided into three groups according to LVEF: ≥ 55% (n = 184); 35%–54% (n = 46); and < 35% (n = 5). In patients with cardiac insufficiency, frequent premature atrial contraction, non-sustained atrial tachycardia, and ventricular arrhythmia were more common. In the LVEF < 35% group, 30% of patients developed non-sustained VT, while the incidences of VT in the EF ≥ 55% group and the EF 35–54% group were 0% and 2%, respectively.These studies were illustrated in Table 4.

**Table 4:** Summary of studies that predict ECG for abnormal cardiac function in patients with DMD

Year	Inclusion criteria	Exclusion criteria	Study type	Sample size	Total ECG	Age enrollment	EF abnormality	ECG abnormality	VT	others
2010	DMD(2004–2009)	None	Retrospective	150	377	10.1–16.9	Not specified	94% DCM with ST changes	27% with VT in DCM, 6 died after 0.68 ± 0.41 years later	5 with abnormal ECG progressed to DCM in 3.7 ± 2.6 years
2013	DMD(2002–2011)	None	Retrospective	155	800	1.8–37.2	Higher V1R and EF < 55%, r = − 0.044	None	None	Higher V1Rdid not predict DCM
2016	DMD(2010–2014)	None	Retrospective	235	442	11.0–17.0	LVEF ≥ 55%	Not specified	0	-
							LVEF = 35%-54%		2%	
							LVEF < 35%		30%	
2018	DMD > 18 years		Retrospective	121	121	18–41	32% in LBBB; 50% without LBBB	Lower LVEF in patients with LBBB	None	–

*Abbreviations*: DMD: Duchenne muscular dystrophy; ECG: electrocardiogram; EF: ejection fraction; LVEF: left ventricular ejection fraction; VT: ventricular tachycardia; LBBB: left bundle branch block; DCM: dilated cardiomyopathy

## Relationship between ECG abnormalities and cardiac function among children with DMD

### Abnormal ECG occurred earlier than cardiac insufficiency in children with DMD

As explained in the above underlying mechanisms of ECG abnormalities in DMD, the absence of dystrophin in the membranes of Purkinje fiber cells may be the pathophysiological basis of early-stage ECG abnormalities in children with DMD. Moreover, abnormal electrical activity may have appeared before cardiomyocyte necrosis and fibrosis, suggesting that ECG changes may be more sensitive to early cardiac involvement in children with DMD, which has been confirmed in many clinical studies. In 2010, a study from the United States [16] reported that the incidence of ECG abnormalities was 44% in DMD patients without DCM. Then, in 2011, another study from the United States [17], which included 78 children < 6 years of age (range 0.2–5.7 years) with normal cardiac function and without hormone treatment found that 78% exhibited at least one ECG abnormality. However, these studies only used LVEF to evaluate cardiac function, which could not truly reflect the early-stage involvement of cardiac function. By applying tissue Doppler imaging (TDI), speckle tracking imaging, multi-modal CMR, to systematically evaluate the cardiac function of children with DMD, and comparing the results with the time to occurrence of ECG abnormalities, it may be possible to more clearly uncover the early-stage cardiac involvement in children with DMD and, thus, formulate more effective early-stage intervention strategies to improve the prognosis of children with DMD.

### Correlation between ECG abnormalities and cardiac function in children with DMD

In 2010, Shah et al. [16] retrospectively analyzed 377 ECG reports from 150 children with myopathy (including 86 children with DMD, with an average age of 10.1–16.9 years) in the United States. They were divided into the DCM group (n = 64) and non-DCM group (n = 86) according to the presence of cardiomyopathy (LVEDD >  + 2 Z score and/or EF < 55%). The results revealed that ECG abnormalities had a certain predictive value for the presence of DCM, with a sensitivity of 95.8% and a specificity of 40.1%. Further subgroup analysis of the types of ECG abnormalities revealed that ST-segment changes, T wave inversion, and Q wave at the inferolateral leads were highly specific for the prediction of DCM (> 95%). Some studies have suggested that a high R wave at lead V1 or right ventricular hypertrophy (RVH) in children with DMD is caused by fibrosis of the posterior basal part of the heart, which suggests that, with the progression of fibrosis, cardiac function is imparied, and RVH should be more apparent on ECG. Theoretically, the ECG manifestations of RVH may be related to cardiac function. Therefore, in 2013, Thrush et al. [66] studied 800 ECG and cardiac ultrasound reports of 155 children with DMD (1.8–37.2 years of age) in the United States, and aimed to explore the relationships of R wave amplitude at lead V1 with LV size and EF. However, it was found that R wave amplitude at lead V1 had no correlation with LV size or EF. Left bundle branch block (LBBB) can lead to uncoordinated ventricular contraction and impaired cardiac function. In 2018, Fayssoil et al. [48] studied 121 DMD patients (18–41 years of age) in France and found that 13% (15/121) had LBBB, and LVEF in the LBBB group was significantly lower than that in the non-LBBB group. More significantly, a study in 2014 [62], including 74 DMD patients, found that the HRV index SDANN (*P* = 0.016) and the fastest heart rate in DCG (*P* = 0.008) demonstrated strong correlations with the index reflecting myocardial fibrosis on CMR imaging (late gadolinium enhancement).

## Predictive value of ECG abnormalities in the prognosis of children with DMD

In 2010, Shah et al. [16] retrospectively analyzed 377 ECG reports from 150 children with myopathy (including 86 children with DMD) in the United States. Among them, 62 children with ECG abnormalities and without DCM were followed up regularly, and 48% (30/62) progressed to DCM, while for the 63 DMD children without ECG abnormalities or color Doppler echocardiography abnormalities in the same period, during the follow-up period, 18% (11/63) had ECG abnormalities, 45% (5/11) progressed to DCM, and ECG abnormalities demonstrated a significant correlation with DCM. In addition, the team also found that VT was a risk factor for death among children with DMD. Among 12 children with VT, 6 died 0.68 ± 0.41 years after VT, 4 died of end-stage heart failure, 1 died of unknown cause, and 1 died of pneumonia and sepsis complications. Among these children with VT, 3 underwent placement of an ICD. After a follow-up of 6.4 ± 3.7 months, they still had paroxysmal VT, but all survived or did not receive additional treatment. In 2018, Fayssoil et al.[48] studied 121 DMD patients (18–41 years of age) in France, 95% of whom needed respiratory support. They found that 34% (41/121) of the patients had RBBB and 13% (15/121) had LBBB. The median follow-up was 6 years. The 5-year cumulative incidence of cardiac events was 17.6%, and the 5-year survival rate was 81.6%. Among cardiac events, 21 patients experienced acute heart failure, 7 from SVT, 3 from VT, and 4 from significant abnormal electrical activity. LBBB was significantly correlated with cardiac events (odds ratio [OR] 12.7 [95% confidence interval [CI] 3.78–42.7) and mortality (OR 4.44 [95% CI 1.44–13.7) in DMD patients. Among the 14 patients with LBBB and heart failure, only those who underwent cardiac resynchronization therapy and ICD placement benefitted, while 6 of the other 12 died.These details were summarized in Table 5.

**Table 5:** Summary of studies that Predictive of ECG abnormalities in the prognosis in patients with DMD

Year	Inclusion criteria	Exclusion criteria	Study type	Sample	Total ECG	Age	Cardiac function	Predict the occurrence of DCM	DCM	Longitudinal Follow-up
2010	DMD (2004–2009)	–	Retrospective	150	377	10.1–16.9	Relevant	Predictable	48%	63 patients had no DCM and no abnormal ECG, and 18% had abnormal ECG. (5/11) of them had subsequent DCM, and the time from abnormal ECG to DCM was 3.7 ± 2.6 years
2018	DMD > 18	–	Retrospective	121	121	18–41	there is an association between LVEF and LBBB, with significantly lower values of LVEF in patients with LBBB as compared to the others	Not mentioned	Not mentioned	Not mentioned

*Abbreviations*: DMD: Duchenne muscular dystrophy; ECG: Electrocardiogram; LVEF: Left ventricular ejection fraction; LBBB: left bundle branch block; DCM: dilated cardiomyopathy

## Conclusion

Although some progress has been achieved in understanding the pathogenesis, diagnosis, and treatment of cardiac involvement in DMD patients in recent years, cardiovascular complications remain the main cause of death in this patient population. The onset of heart injury in children with DMD is inconspicuous, and the prognosis is poor once it develops to the stage of heart failure. Research has revealed that before inflammation, necrosis, and fibrosis of cardiomyocytes in DMD patients, Purkinje fiber cells undergo changes due to dystrophin deletion, which provides important pathophysiological evidence that ECG may more sensitively reflect and/or detect early manifestations of myocardial involvement in DMD patients compared with echocardiography and even CMR imaging. Relevant clinical studies have also confirmed this inference (i.e., significant abnormal ECG changes already exist in DMD patients before cardiomegaly and/or LVEF decrease). Due to varying sample sizes, inclusion criteria, and population backgrounds across studies, the types and incidences of ECG abnormalities in children with DMD were quite different. However, several consistent findings were observed. Pathological Q waves at leads I, II, III, avL, avF, and V4-V6, increased R wave amplitude with increased R/S ratio at lead V1, RVH, left ventricular high-voltage, and ST-segment and T wave changes were common abnormal ECG changes in children with DMD. Sinus tachycardia and short PR interval were common and characteristic ECG abnormalities in children with DMD. Conduction block, including CRBBB, CLBBB, and cAVB, was not common in children with DMD, and most occurred in older children with DMD. QT interval prolongation was rare. SVT and VT may occur; however, most occur in older children and/or DMD patients with clinical symptoms of cardiac insufficiency and/or impaired cardiac function. Patients with DMD may exhibit HRV changes in the early stage(s) of the disease, with dysfunction of cardiac autonomic regulation, decreased vagal tone activity, and increased sympathetic tone activity. With increases in age and decreases in cardiac function, the proportion of ECG abnormalities in DMD patients increase significantly. However, the correlation between dystrophin gene mutation and ECG abnormalities has not been determined and warrants further investigation. Some characteristic ECG changes, such as ST-segment changes, T wave inversion, Q wave at the inferolateral leads, LBBB and SDANN, have a certain correlation with the indexes of cardiac remodeling or impaired cardiac function in DMD patients, while VT and LBBB have demonstrated relatively good predictive value for the occurrence of long-term DCM and/or adverse cardiovascular events or even death in DMD patients.

However, most current investigations are limited to retrospective descriptive studies with small sample sizes, and there is little research investigating DCG; as such, there is a lack of data regarding electrocardiovector changes in DMD patients. Therefore, further larger prospective longitudinal studies in broader age range warrant to be carried out to uncover the characteristics of general ECG, dynamic ECG and vectorcardiogram, to verify the role of ECG in detecting early cardiac injury and in predicting the worse outcomes in patients with DMD.

## Data Availability

Data sharing not applicable to this article as no datasets were generated or analysed during the current study.

## Abbreviations

DMD: Duchenne muscular dystrophy
ECG: Electrocardiogram
EF: Ejection fraction
LVEF: Left ventricular ejection fraction;
LBBB: Left bundle branch block
CLBBB: Complete left branch bundle block
CRBBB: Complete right bundle branch block
CMR: Cardiac magnetic resonance
CXMD: Canine X-linked muscular dystrophy
cAVB: Complete atrioventricular block
SVT: Supraventricular tachycardia
VT: Ventricular tachycardia
DCG: Dynamic ECG
DCM: Dilated cardiomyopathy
ICD: Implantable cardioverter-defibrillator
HRV: Heart rate variability
SDNN: Standard deviation of all normal-to-normal (NN) intervals
SDANN: Standard deviation of the averages of NN intervals in all 5 min segments of the entire recording
RMSSD: Square root of the mean of the sum of the squares of differences between adjacent NN interval
pNN50: Percentage difference between adjacent NN intervals that are greater than 50 ms
HF: High frequency power
HF.nu: High frequency power normalized unit
LF/HF: Ratio of low–high frequency power
RVH: Right ventricular hypertrophy
OR: Odds ratio
